# Supplementary material for: Role of Neuron–Glia Signaling in Regulation of Retinal Vascular Tone in Rats
Source: Int J Mol Sci. 2019 Apr 20;20(8):1952. doi: 10.3390/ijms20081952 (PMC6514555; doi:10.3390/ijms20081952)
Supplement: Supplementary file 1 [file ijms-20-01952-s001.pdf]

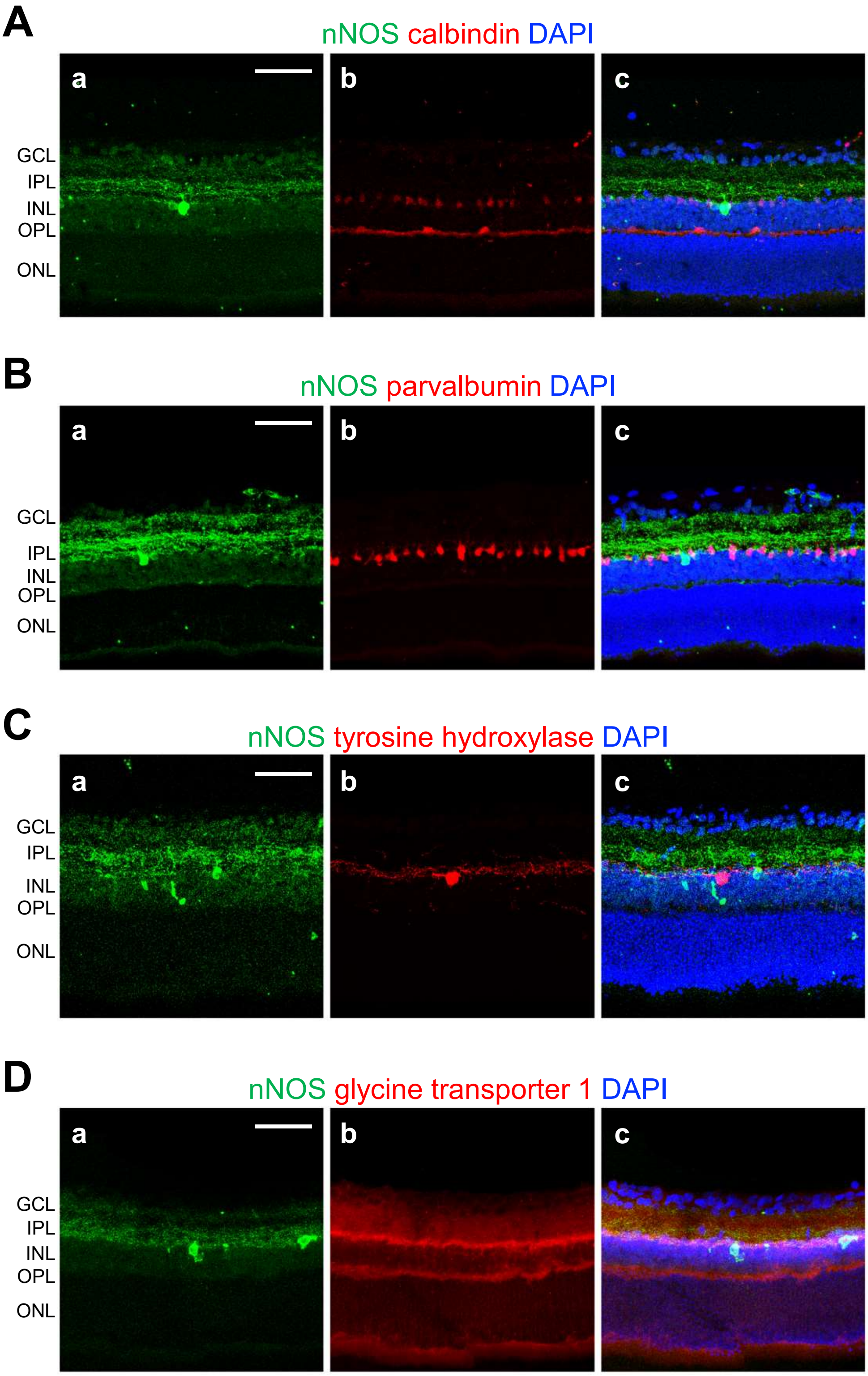

Supplementary Figure 1. Co-labeling of neuronal NO synthase (nNOS) with amacrine cell markers in the retina. Confocal microscopy images of retinal cross-sections labeled with anti-nNOS and anti-calbindin-D-28K (A), anti- parvalbumin (B), anti-tyrosine hydroxylase (C), and anti-glycine transporter 1 (D). The co-localization of nNOS immunoreactivities with glycine transporter 1-, tyrosine hydroxylase-, parvalbumin-, calbindin-D-28K-positive cells were not detected. Scale bar: 50 μm. DAPI, 4',6-diamidino-2-phenylindole; IPL, inner plexiform layer; OPL, outer plexiform layer; ONL, outer nuclear layer.
